# Entangled Time Hops: Doomsday Clocks, Pandemics, and Qualitative Research’s Responsibility

**DOI:** 10.1177/1077800420960188

**Published:** 2021-09

**Authors:** Stephanie Anne Shelton

**Affiliations:** 1 The University of Alabama, Tuscaloosa, USA

**Keywords:** pandemic, COVID-19, racism, qualitative research, stories, responsibility

## Abstract

This article explores the micro- and macro-level implications of the dual global pandemics of COVID-19 and racism through a narrative structure based on Barad’s discussion of “timehops.” Weaving personal, national, and international stories, the article explores qualitative research’s responsibility and potential to offer new ways to respond to the entanglements of people, places, moments, materials, and these pandemics.

When quarantine started months ago in response to COVID-19’s spread, I found time slipping away. Trying to trace and track time was like trying to hold onto water: messy remnants marked my efforts, but my hands remained empty. Clocks, which had seemed so faithful suddenly held little meaning except to alert me to another online meeting. The linearity of the day was no longer a line but a scribble that looped, broke, and collapsed on itself. In my struggle to mark out my days, I embarked on a new daily adventure: I joined a writing community that engaged in daily prompts examining microscopic and macroscopic effects of the pandemic (Markham & Harris, 2020). This new connection did not make telling/marking time any easier, but it made the elasticity of time more meaningful as I engaged in writing/creating/dreaming with others around the world.

## Chaotic Counting: A Doomsday Clock and Peace Clock

Time’s new messiness necessitated my considering how it might be understood and traveled differently, in my personal setting(s), nationally, and globally. In reflecting on the United States 1945 nuclear destruction of Hiroshima, Japan, Karen Barad describes the creations of both a Doomsday Clock and Peace Watch Clock. The Doomsday Clock’s function is to provide an “estimation of our proximity to global catastrophe” (Barad, 2017, p. 57). Conversely, the Peace Clock “is synchronised to peace instead of war” (p. 59), and restarts each time that there is a nuclear weapon test. The two clocks, regularly reset based on human activities, work to measure how close humankind is to achieving world destruction or peace.

Clocks whose movements are based on human actions. They trouble “the nature of time and being, or rather, time-being” (p. 62) by examining the simultaneity of impending apocalypse and harmony, based not on the predictable crunch of clock gears but on people’s stumbling, capricious efforts. To reflect this forward/backward, countdown/up, this article will move in what Barad calls “travel hops” (p. 69)—different moments in different spaces, with the micro of personal experiences linked with macro across the United States and the world. Although seemingly disparate, “[t]hese stories inhabit each other—a strange topology that already anticipates the kind of temporal imaginaries” that time hopping works to explore (p. 62). Through this temporal fluidity, this article draws on prompts/musings from the writing community’s process(es) to explore the dual pandemics of COVID-19 and racism; it concludes with considerations of how qualitative researchers might approach these intertwined pandemics with “an ongoing responsiveness to the entanglements of self and other, here and there, now and then” (Barad, 2007, p. 394).

### Time Hops: The Dancing Pot and a Global/National Pressure Cooker

#### 1987, A tiny, crooked house

My siblings and I watch Mama cook with a dented silver pressure cooker. Before each meal, the pot shimmies and hisses on the stove, its pressure valve rotating round and round in manic, clattering desperation. The warped and uneven kitchen floors leave the stove slightly akimbo. The combination of sloping floors, unlevel cooking surface, and dancing pot make for adventurous food preparation. We run through the house laughing and playing, and suddenly Mama shouts, “STOP RUNNING! The pot will BLOW UP.” Breathlessly, we freeze in place, horrified at being killed by a kitchen appliance. “I want to be killed by a dinosaur,” my brother whisperingly confides.

#### 2020, news headlines

“Amid protests and a pandemic, what does it mean to be American in 2020?” (Dastagir, 2020).

“A World on Fire: Here are All the Major Protests Happening around the Globe” (Kaplan et al., 2020).

#### 2020, worldwide

Each day, I turn on the news and see that pot again shaking and bubbling, just barely not exploding across the United States and world. The protestors move through uneven, slanting streets—energy and motion shaking the asphalt beneath them (Taylor, 2020). The literal ground quivers, vibrating with anticipation.

The Peace Clock clicks forward, hoping for resolution, equity, and justice.

The Doomsday Clock continues to countdown, expecting destruction.

#### 1985, A tiny, crooked house

When the pot finishes cooking, Mama releases the pressure valve and the pot sigh-hisses a wet, hazy steam that fills the kitchen. “Has it blown up?” we ask, concerned by the racket and smoke. “No,” Mama assures, “I’m just releasing the pressure. If I hadn’t, it would explode, though.”

### Time Hops: I Can’t Breathe

#### 2010, Sandersville, GA, USA

“You’re late to class. *Where* have you been?” The high school student presses his back and his right palm against the wall, breathing in gulps. His eyes are filled with fear; perspiration shines on his forehead and cheeks.

“Hold on, Ms. Shelton,” he wheezes, “I can’t breathe good yet.”

I wait, my annoyance replaced with concern. “Do you need to sit down?” I ask.

He shakes his head “No”; his palm leaves the wall to cover his pounding heart. After another minute, his breathing is less labored.

Again I press, “Where have you been? What happened?”

He grimaces.

“Man, Ms. Shelton, that security officer was in the halls looking for folks to get, and I was on my phone because my Daddy just got arrested. I was finding out was he okay. I knew I was gonna be in big trouble: skipping class, bringing my phone to school, using my phone. I couldn’t let him get me. Who knows what would’ve happened?”

The question stretches into my thoughts of an armed school security guard and a lone Black boy in the hallway.

I do not take his phone; I do not send him to the discipline office.

I point to the water fountain, asking, “Do you need some water?”

Still gasping for air, he nods and pushes his body from the wall.

#### July 17, 2014, Staten Island, NY, USA

Eleven times Eric Garner says, “I can’t breathe” as a White officer uses a chokehold. He has been accused of selling cigarettes without tax stamps. He is murdered. The officer is not charged (BBC News, 2019).

#### 2020, worldwide

“The pneumonia that COVID-19 causes tends to take hold in both lungs. Air sacs in the lungs fill with fluid, limiting their ability to take in oxygen” (Johns Hopkins Medicine, 2020).

#### May 25, 2020, Minneapolis, MN, USA

For 8 min and 46 s, George Floyd pleads, “I can’t breathe,” as a White officer kneels on his throat. He had been accused of trying to buy cigarettes with counterfeit money. He is murdered. After over a week of protests across the nation and globe, the four officers—the one who knelt on this throat and three who watched—are charged (Singh, 2020).

#### 2020, worldwide

“Bibasilar crackles are a bubbling or crackling sound originating from the base of the lungs. They may occur when the lungs inflate or deflate.” These are sounds of COVID-19, as a patient struggles to breathe (Stephens, 2020).

Around the world, people cannot breathe. Some as they fight to survive the COVID pandemic, others to survive racism and White Supremacy. All wear fear in their eyes and a sheen of perspiration on their faces, gasping for air.

The Peace Clock stops. Restarts.

The Doomsday Clock accelerates.

### Time Hops: Humanizing Numbers

#### May 6, 2020, Tuscaloosa, AL, USA

I scroll down my phone, scanning for updated numbers. Yesterday there were 95 new COVID cases in the state. I gasp. Today there are 412 (Alabama Public Health, 2020).

#### February 23, 2020, Worldwide

The World Health Organization (WHO, 2020b) states that COVID-19 is an international public health emergency but not a global pandemic. It is not yet a pandemic, says WHO Director-General Ghebreyesus, because the world is “not witnessing the uncontained global spread of this virus, and we are not witnessing large-scale severe disease or deaths” (n.p.).

#### February 27, 2020, 4 days later, Washington, DC, USA

During a press conference on the globally growing number of COVID-19 cases, President Donald Trump states, “When you have 15 [U.S.] people [with Coronavirus], and the 15 within a couple of days is going to be down to close to zero, that’s a pretty good job we’ve done” (The White House, 2020, n.p.).

#### April 28, 2020, Tuscaloosa, AL, USA

In addition to the surging local cases, the United States has surpassed 1 million cases of COVID-19 and more than 57,000 deaths (NPR, 2020). The United States has more cases than any other country in the world.

#### February 28, 2020, 2 months earlier, Washington, DC, USA

Discussing coronavirus, Donald Trump says, “It’s going to disappear. One day, it’s like a miracle, it will disappear” (Goldberg, 2020).

#### August 16, 2020, worldwide and USA

There are 21.5 million confirmed coronavirus cases and 772,000 deaths. The United States ranks #1 in both categories, with 5.37 million cases and 169,000 deaths (Google COVID-19 Alert, 2020).

#### August 1, 2020, Tuscaloosa, AL, USA

My phone tells me that there are 3,873 local cases and 63 deaths—in the state where there are nearly 88,000 cases and nearly 1,600 deaths (Google COVID-19 Alert, 2020). Just across the state border, in Georgia where Mama lives, there are more than 168,000 cases and nearly 3,600 deaths. In Mama’s rural county, there are 400 cases, and today marked the second death.

“I don’t like how these numbers make us forget people. People and their families and friends,” Mama tells me on the phone. The recent death was someone she knew well. “He was a good person. He did a lot of community outreach and helped others. That ‘2’ doesn’t say any of that. And, I don’t know who the ‘1’ was, but they mattered, too.”

#### May 2020, USA

On May 24, 2020, the United States surpasses 100,000 COVID-related deaths. *The New York Times* runs a feature naming them and sharing brief biographical information (“An Incalculable Loss,” 2020). Marion Kruger, from Kirkland, Washington, was a “great-grandmother with an easy laugh,” George Freeman Winfield, from Shelburne, Vermont, “could make anything grow,” and Romi Cohn, from New York City, New York, “saved 56 Jewish families from the Gestapo.” Without this humanizing news coverage, they become only numbers.

Less than a week later, on May 30, people gather in a park near my home to protest police brutality, racist violence, and the murders of George Floyd, Eric Garner, Breonna Taylor, and so many others. Protestors wear and hand out masks, to help counter one pandemic while protesting the other (Pillion, 2020). Our local action is echoed around the nation and world, as millions take to the streets to demand change.

#### August 4, 2020

During an interview, *Axios* reporter Jonathan Swan asks Donald Trump about the fact that—daily—1,000 United States citizens die from COVID-19. Trump replies, “It is what it is” (Shabad, 2020).

The Doomsday Clock continues counting down, death and apathy for death fueling it.

The Peace Clock moves forward, powered by the empathy, hope, and protests reverberating around the globe.

## Responsibility and a Third Clock

The time hops travel through decades, through cities and nations, through hope and fear, through resolve and anger. The Doomsday and Peace Clocks’ either/or measurements are inadequate for these both/and moments and the human pulses powering them. The two clocks, measuring human (in)actions rather than minutes, operate based on clear starts and stops; their motions begin/halt/resume, relative to concrete moments.

Though different from one another, each of these clocks’ times treats time as determinant and singular; in essence, each clock has a pointer pointing at a single position on a clock face and marks one time at a time. (Barad, 2017, p. 60)

They are useful reminders of the degrees to which actions shape moments and emotions, but they are inadequate in exploring/crafting a “radical rethinking of the nature of time” (p. 60) within these pandemics. It is time for a third clock, one whose gears run not on absolute destruction or utopic peace, but on everyday connections. One whose hands measure out intra-active responsibility as means to navigate and move alongside, rather than away from, the past and the present.

This is an Intra-active Clock.

Intra-action “signifies the mutual constitution of entangled agencies [. . .] Intra-action constitutes a radical reworking of the traditional notion of causality” (Barad, 2007, p. 33). An Intra-active Clock rejects the Doomsday and Peace Clocks’ causation mechanisms; rather than counting down/up, this third clock counts with/in and alongside. Its gears flow rather than clank, with motions powered by “relationality and aliveness (vitality, dynamism, agency)” (p. 33). Its fluidity disrupts boundaries between people, places, and moments; these “entanglements of self and other, here and there, now and then” (p. 394) produce new possibilities to understand and navigate the micro- and macro- of the viral infections of COVID and bigotry.

### Intra-Active Responsibility

A key component of intra-action is responsibility because these multiple “entanglements require/inspire a different sense of a-count-ability, [. . .] a different calculus of response-ability” (Barad, 2014, p. 178). These dual pandemics have intertwined the world in new and important ways. Nearly 200 nations are collaborating to produce affordable COVID-19 vaccinations (WHO, 2020a), while simultaneous protests sparked by anti-Black violence in the United States have brought together millions across dozens of nations (Cave et al., 2020). Intra-active responsibility, such as these instances that have connected so many across multiple spaces and moments, is “about making connections and commitments” (Barad, 2007, p. 392). Even more specifically, this relational understanding of responsibility requires “a praxis of care and response” (Haraway, 2016, p. 105) that moves beyond individuality to “a relation of responsibility to the other” (Barad, 2007, p. 391).

#### Responsibility, Stories, and New Possibilities

As the Intra-active Clock flows and weaves, moving with these relational enmeshments, it does not use digital or analog mechanisms to travel time; rather, stories power its gears. Both Barad and Haraway, whose work influenced Barad, noted that attending to everyday stories provokes “quite definitive response-abilities that are strengthened in such stories” (Haraway, 2016, p. 115). Within this world and these pandemics, we “do have a role to play” (Barad, 2007, p. 172), and these stories offer means to take up that role. My time hops afford opportunities to creatively examine the ways that micro-level moments in my life, such as a shimmying pot and terrified high school student, connect to macro-level instances of viral infection and protest, despite separations of years and miles.

Qualitative research has long embraced the possibilities afforded through storying. Denzin (2019) emphasized “a call to action” for qualitative social justice (p. 19) where stories, through methods such as autoethnography, drama, narrative, and poetry, invite “self-other interactions” (p. 20). These stories tap into “the ‘restorative function of qualitative methodological techniques’” (p. 38) and offer opportunities to “confirm social injustice,” to celebrate efforts for justice, and to care about the ways that we are all tied to one another.

Turning back to the Intra-active Clock, its slippery operations offer spaces for such stories to exist, to be taken up, to matter. These stories, like the Clock, are not bound to specific individuals/locations/moments; they flow across/within all, enmeshing people, space, time, and materials to explore and explode what was previously assumed possible. Within and beyond these pandemics, “the very nature and possibilities for change are reworked” (Barad, 2007, p. 393) through these stories and the responsibility that they require/offer through/within/because of our many entanglements. (Re)writing stories in the face of dual pandemics offers opportunity to “honor our responsibility by helping to shape the future” (p. 396). In the face of millions of deaths worldwide, now six in Mama’s town, due to COVID and weaponized racism, our stories offer chances “to come to terms with the infinite depths of our inhumanity and out of the resulting devastation, to nourish the infinitely rich ground of possibilities” for living, writing, connecting differently (Barad, 2017, p. 86). It is time.
